# Functional roles for myosin 1c in cellular signaling pathways

**DOI:** 10.1016/j.cellsig.2012.09.026

**Published:** 2013-01

**Authors:** Lisa M. Bond, Hemma Brandstaetter, John Kendrick-Jones, Folma Buss

**Affiliations:** a Cambridge Institute for Medical Research, University of Cambridge, Wellcome Trust/MRC Building, Hills Road, Cambridge, CB2 0XY, United Kingdom; b MRC Laboratory of Molecular Biology, Hills Road, Cambridge, CB2 0QH, United Kingdom

**Keywords:** Myosin, Signaling, Lipid raft

## Abstract

Cellular signaling pathways underlie the transfer of information throughout the cell and to adjoining cells and so govern most critical cellular functions. Increasing evidence points to the molecular motor myosin 1c as a prominent player in many signaling cascades, from the integrin-dependent signaling involved in cell migration to the signaling events underlying insulin resistance. Myosin 1c functions on these pathways both via an important role in regulating lipid raft recycling and also via direct involvement in signaling cascades. This review provides an overview of the functional involvement of myosin 1c in cellular signaling and discusses the possible potential for myosin 1c as a target for drug-based treatments for human diseases.

## Highlights

► Myosin 1c is a prominent player in many signal transduction pathways. ► Lipid raft trafficking and recycling is mediated by myosin 1c. ► The distribution of lipid rafts determine the spatial regulation of signaling events. ► Myosin 1c is a potential drug target for signaling-related diseases.

## 1 Introduction

The regulation of cellular processes relies on dynamic communication between a given cell and its surroundings. This communication often occurs via exposure to chemical signals (e.g. hormones, cytokines) that bind membrane receptor proteins and trigger intracellular signal transduction cascades. These signaling pathways are carefully coordinated, interconnected and highly conserved networks of kinase effector proteins and secondary messengers that amplify and integrate external signals to produce distinct cellular responses (e.g. changes in gene expression). In this manner, signaling cascades regulate a wide range of fundamental processes, from cellular growth, differentiation, and apoptosis to migration and the immune response [1]. Some commonly studied signal transduction pathways are the multichain immune recognition receptor (MIR) pathways mediating immune response to specific antigens/antibodies [2], the mitogen-activated protein (MAP) kinase and Wnt signaling pathways regulating development [3,4], and the insulin signaling pathway regulating blood glucose levels [5].

As in the case of most pathways involving the regulated transmission of a signal or other cargo throughout the cell, signal transduction pathways are often associated with the myosin protein family. Myosin proteins are a class of molecular motors that traditionally use the energy derived from ATP hydrolysis to transport cargo along actin filaments or tether cargo at specific intracellular locations [6]. These myosin motors mediate many key cellular processes such as motility, cytokinesis, organization of the actin cytoskeleton and, notably, signal transduction. For example, myosins in class III are themselves protein kinases that function directly in phototransduction signal cascades [7]. Furthermore, myosin IX proteins serve as GTPase activating proteins (GAPs) that negatively regulate Rho GTPases and so directly influence Rho signaling pathways involved in actin remodeling/cell migration [7–9]; whereas myosin VI itself is phosphorylated by p21-activated kinase (PAK) and has been suggested to play a role in a PAK signaling pathway mediating membrane ruffling in response to epidermal growth factor stimulation [10]. In addition, nonmuscle myosin II directs the cell surface localization of receptors for inositol (1,4,5)-trisphosphate [11] and regulates the function of the receptor tyrosine kinase discoidin domain receptor 1 (DDR1) in a signaling network effecting cell migration/spreading [12]. Myosin IIA also plays a role in regulating the endocytosis of the cytokine receptor CXCR4 and subsequent onset of stromal cell-derived factor-1 alpha (SDF-1 alpha) signaling [13], and finally modulates the internalization and downstream signaling pathways of the epidermal growth factor receptor (EGFR) [14].

Recent insight into the intracellular roles of myosin 1c highlights this myosin as another critical player in the field of cell signaling. Myosin 1c is a single-headed, low duty ratio motor with a three-part domain structure similar to that found in most myosin proteins: it has an N-terminal motor domain that binds to actin and hydrolyzes ATP, a neck (lever-arm) region that binds to three regulatory calmodulin light chains, and a tail domain with a pleckstrin homology (PH) lipid-binding motif [15–17]. The motor localizes to the plasma membrane and actin-rich structures near the cell surface (e.g. ruffles/lamellipodia) as well as the cell nucleus and lipid membranes throughout the cell [18,19]. This review will highlight the broad impact of myosin 1c on cell signaling by discussing the involvement of this myosin in many different intracellular signaling pathways. The therapeutic implications of this involvement for myosin 1c in cell signaling will then be explored.

## 2 Overview of cellular functions of myosin 1c

Examining the distinguishing structural and kinetic features of myosin 1c provides insights into its general functions within the cell. Myosin 1c is a ‘short-tailed’ member of the myosin I family, containing only the first of the three existing myosin I tail homology domains. Such short-tailed myosins are typified by slower ATPase rates than their long-tailed counterparts in the myosin I family and thus have been classified as slow ‘strain sensor’ myosins [20]. These myosin proteins appear to have been designed either to transport/anchor heavy cargo or to crosslink the stress-bearing actin filaments required for tethering or maintenance of intracellular cortical tension [21,22]. This type of tethering/tension modulating role for myosin 1c is further supported by the observation that this myosin has a two-phase power stroke and so is well adapted for maintaining significant tension without continuous dissociation from (and reassociation with) actin filaments [23].

Functional studies of myosin 1c indicate that the motor utilizes its tension modulating/tethering capacities in a variety of critical cellular processes. On the exocytic pathway, myosin 1c has been implicated in the regulated secretion of glucose transporter 4 (GLUT4)-containing vesicles [24,25]. Myosin 1c has also been shown to couple polymerizing actin filaments to membranes to modulate force production during compensatory endocytosis [26]. Furthermore, myosin 1c has been implicated in the transport of aquaporin-2 (AQP2)-containing membrane vesicles to the apical region of renal collecting duct cells [27], and is required to maintain the correct localization of the slit diaphragm protein Neph1 at the podocyte membrane [28] and the proper localization of sodium channels at collecting duct cell membranes [29].

In addition to these roles in intracellular trafficking, myosin 1c has been widely implicated in the maintenance and organization of actin rich structures at the cell periphery: for example, in the dynamic rearrangement of the cortical actin network required for membrane ruffle formation in adipocytes [25,30]. In motile cells, myosin 1c plays a role in lamellipodia dynamics [31], in the turning of growth cones to direct neuronal cell motility [32], and in the transport of G-actin to the leading edge of migrating epithelial cells [33]. Myosin 1c is also implicated in cytoskeletal rearrangements within the lamellipodia and filipodia of spreading B-cells [34]. On a more divergent functional note, a splice variant of myosin 1c with an N-terminal extension is targeted to the nucleus and speculated to play a role in transcription and other nuclear processes [19].

## 3 Myosin 1c in cell signaling: lipid raft regulation

Insight into the distinct molecular role of myosin 1c common to all of these dynamic processes has been greatly enhanced by the recent discovery that myosin 1c regulates lipid raft trafficking and recycling [35]. This function likely underlies many of the cellular roles for myosin 1c discussed above. As lipid rafts are intimately linked to cellular signaling pathways, this discovery also targets myosin 1c as a major player in intracellular signaling. This section will provide an overview of the raft-based involvement of myosin 1c in cell signaling.

### 3.1 Lipid rafts and cell signaling

Over the last fifteen years, the concept of lipid rafts has drastically revised traditional theories of a passive and freely diffusing lipid-protein cell membrane. These lipid rafts are membrane microdomains characterized by a concentration of sphingolipids and cholesterol that compartmentalize cellular processes by permitting the ordered sorting and concentration of specific proteins within small membrane regions [36,37]. Rafts were originally characterized by their resistance to extraction with non-ionic detergents, but recent advances in optical techniques have made it possible to visually confirm their presence [38]. The cohesiveness of a given lipid raft is based on lateral associations between its lipid components [36] and such cohesive regions of ordered sphingolipid/cholesterol membrane have been identified in trafficking pathways not only at the plasma membrane and on the endocytic pathway, but even in the early stage of secretory transport between the endoplasmic reticulum and the Golgi complex [39]. During their transport through the secretory sorting pathways, lipid rafts selectively attract and so compartmentalize specific cellular proteins, particularly glycosylphosphatidylinositol (GPI)-anchored proteins and certain fatty acylated (palmitoylated/farnesylated/myristoylated) proteins [36,40]. The resulting clustering of proteins at outer membranes is modulated and stabilized by the actin cytoskeleton [41] and forms the basis for the functional grouping of proteins during a wide range of processes, from cell migration and intracellular trafficking [42] to embryonic stem cell renewal [43] and signal transduction [44].

Most pertinent to this review is the widespread function of lipid rafts in signal transduction processes. The value of lipid rafts in facilitating signal transduction is clear: a raft provides a microenvironment for localized signaling processes, permitting the grouping of signaling receptors and associated signaling proteins in an isolated and concentrated region [44]. In particular, isolated raft regions attract individual signaling receptors (many of which are GPI-anchored, e.g. CD14) and selectively recruit associated signaling kinases and guanosine triphosphatases (GTPases) (e.g. dually acylated Src kinases and the palmitoylated/farnesylated GTPase H-Ras), while actively excluding proteins such as membrane phosphatases that could interfere with signal transduction [44,45]. A cell can thus regulate signal transduction by modifying the post-translational processing of signaling proteins to change their recruitment to lipid rafts. When one considers the regulation capabilities afforded by the fact that certain signaling proteins only associate with lipid rafts when activated and that proper signal transduction in certain GPI-anchored proteins may actually depend on raft association [44,46], the full value of lipid rafts as the functional organizers of cellular signaling becomes clear. It is not surprising then that lipid rafts have been linked to a wide variety of intracellular signaling processes, including T-cell antigen receptor signaling, Ras signaling, immunoglobulin E signaling, and tumor necrosis factor receptor (TNFR) signaling [45,47]. In short, the spatial segregation afforded by lipid rafts facilitates the efficiency and regulation of signaling processes throughout the cell.

### 3.2 Myosin 1c regulates lipid raft trafficking

Previous findings that myosin 1c binds to PI(4,5)P2, a phospholipid enriched in lipid rafts [17], and localizes to lipid rafts in podocytes [28] set the stage for a recent study probing the general relationship between myosin 1c and lipid rafts [35]. In this study, Brandstaetter et al. confirm the spatial association of myosin 1c with lipid rafts by demonstrating that myosin 1c colocalizes with GPI-anchored raft markers at the plasma membrane and on intracellular recycling tubules in live cells, as well as co-fractionates with raft markers in a flotation assay. The study then demonstrates that siRNA knockdown of myosin 1c reduces levels of lipid raft-linked proteins at the plasma membrane (and concentrates these proteins in a perinuclear recycling compartment), while overexpression of myosin 1c increases the proportion of lipid rafts at the plasma membrane. The functional role thus indicated for myosin 1c in the exocytosis of lipid rafts from the perinuclear recycling compartment is further supported by the demonstration that myosin 1c knockdown specifically reduces the number of cells containing exocytic lipid raft tubules emanating from this recycling compartment. Brandstaetter et al. speculate that myosin 1c may regulate lipid raft recycling by modulating the sorting of specific protein cargo into lipid rafts at the recycling endosome or by using the tension of the actin cytoskeleton to promote tubule formation at the recycling endosome and fusion of lipid rafts with the plasma membrane. The new role for myosin 1c as a regulator of the intracellular trafficking (and potentially protein composition) of the lipid rafts crucial for proper signal transduction highlights myosin 1c as a broad scale regulator of cellular signaling.

The following two subsections provide a detailed discussion of the signaling pathways in which we now have direct evidence that myosin 1c functions via lipid raft regulation.

### 3.3 Myosin 1c as a regulator of integrin-mediated signaling pathways involved in cell spreading and migration

Cells characteristically spread on an extracellular matrix by extending membrane protrusions such as filopodia and lamellipodia/membrane ruffles, and adhere to their surroundings at contact sites called focal adhesions [48]. Repetition of this spreading and adhesion process in one part of the cell while simultaneously removing adhesion sites on opposing sides of the cell forms the basis for directed cellular migration [49]. The processes of cell spreading and migration intimately rely on the integrin receptor, a transmembrane protein that clusters at adhesion sites to permit communication between the extracellular matrix and interior of the cell. In particular, these integrins mediate signal transduction events regulating actin organization and adhesion assembly [50]. For example, a specific signal can trigger integrins to activate the tyrosine kinase FAK (focal adhesion kinase), which facilitates activation of the tyrosine kinase Src. The binding of Src to the protein p130Cas recruits the signaling adaptor Crk, which binds p130Cas. This mediates the activation of the Rho GTPase Rac1, which operates with the GTPase Cdc42 to activate the WASP/WAVE family of Arp2/3 complex activators and thereby induce the actin polymerization/branching that underlies lamellipodia and filopodia extension during spreading and migration [50,51].

Like many signaling events, these integrin-mediated signaling pathways regulating cell migration and spreading rely on lipid rafts [52], which have been shown to recruit critical signaling molecules such as Rac and Cdc42 to the plasma membrane [53]. This recruitment is specifically triggered by integrin engagement, and the disruption of raft targeting results in a loss of downstream signaling [53]. Rafts have also been cited for their ability to concentrate the signaling molecules necessary for migration/spreading, thereby promoting efficient and rapid signal transduction [54]. For example, Src kinases are activated within lipid rafts [55] and the Rho kinases mediating focal adhesion formation cluster with Src in these rafts [56]. In fact, intact rafts are required for prolonged activation of Rac and actin polymerization [57]. Furthermore, the differential sorting of signaling molecules into lipid rafts at the front versus rear of the cell permits the spatial segregation of directed forward movement [54]. The overall importance of lipid rafts in these signaling processes is further supported by the observation that disruption of lipid rafts completely abolishes lamellipodia formation and migration in MCF-7 cells [54].

**Fig. 1:**
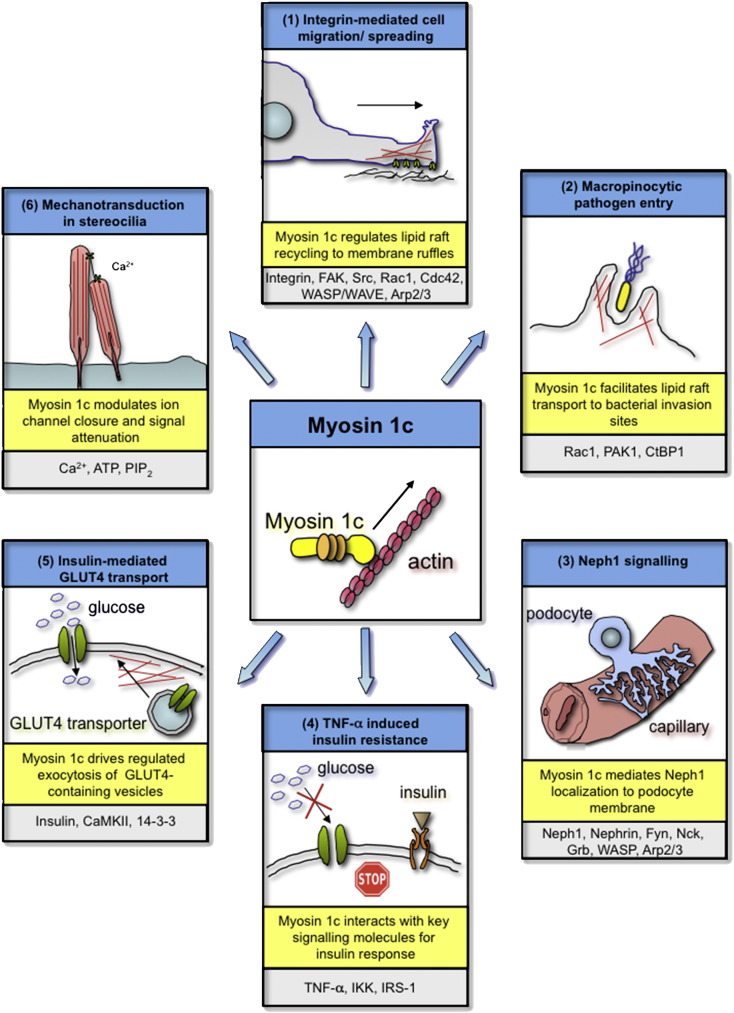
Myosin 1c in cellular signaling pathways. This figure provides an overview of the cellular signaling pathways involving myosin 1c function. In particular, myosin 1c has been shown to play a role in (1) the integrin-mediated signaling pathways underlying cell migration, (2) the signaling events facilitating pathogen uptake by macropinocytosis, (3) the Neph1 translocation and signaling mediating podocyte foot formation, (4) the tumor necrosis factor-alpha (TNF-α)-induced insulin resistance pathways, (5) insulin-stimulated GLUT4 transport to the plasma membrane, and (6) mechanical signal transduction in the hair cells of the inner ear. The molecular role of myosin 1c in each pathway is outlined and additional key signaling players are displayed for reference. This presentation of the diverse assembly of signaling pathways involving myosin 1c function highlights the importance of this motor in cellular signaling. Abbreviations: FAK (focal adhesion kinase), PAK1 (p21-activated kinase 1), CtBP1 (C-terminal binding protein 1), Nck (non-catalytic region of tyrosine kinase adaptor protein 1), Grb2 (growth factor receptor-bound protein 2), IKK (IκB kinase), IRS-1 (insulin receptor substrate 1), CaMKII (Ca^2 +^/calmodulin-dependent protein kinase II), Ca^2 +^ (Calcium), ATP (adenosine triphosphate), and PIP_2_ (phosphatidylinositol 4,5-bisphosphate).

The role played by myosin 1c in the regulation of lipid raft recycling indicates that this motor protein also regulates the integrin signaling pathways underlying cell migration and spreading. A lipid raft based role for myosin 1c in these cellular signaling pathways is reinforced by the observation that knockdown of myosin 1c dramatically reduces cell spreading and causes a reduction in lipid raft markers at the cell surface of spreading HeLa cells [35]. Myosin 1c knockdown also results in the redistribution of focal adhesions, as well as a reduction in the migration speed and track length of migrating cells [28,35]. These findings thus indicate a clear role for myosin 1c in mediating integrin-based signaling pathways via a regulation of lipid raft recycling (Fig. 1 (1)).

### 3.4 Myosin 1c as a regulator of the signaling pathways involved in macropinocytic pathogen entry

A virus or bacteria entering a given cell may use the cell's own endocytic machinery to bypass the plasma membrane. In particular, an increasing number of studies show that many pathogens infect a cell via uptake into macropinosomes, large vesicles formed by membrane ruffling that traditionally function in fluid uptake [58,59]. The entire process of macropinocytosis relies on activation from an external signal, generally stimulation by a growth factor (e.g. epidermal growth factor) or tumor promoting factor (e.g. phorbol myristate acetate) [59]. In addition to the integrin-based signaling pathways for actin regulation discussed above, the entry of different pathogens by macropinocytosis involves a diverse set of signaling pathways, each with their own characteristic collection of molecular players [58]. For example, the uptake of vaccinia virus by macropinocytosis activates a signaling cascade including the GTPases Rac1 and RhoA, protein kinase C, and the membrane protein and kinase substrate ezrin [60]. This signaling cascade specifically forms the actin and ezrin containing cell protrusions necessary for vaccinia virus entry [60]. In another example, the closure of macropinosomes during the uptake of adenovirus 3 requires a signaling cascade in which the Rac1 GTPase activates PAK1 (p21-activated kinase 1) and PAK1 subsequently activates CtBP1 (C-terminal binding protein 1) [58,61].

As one might expect, lipid rafts are also implicated in the signaling processes involved in macropinosome formation for pathogen entry [62,63]. Spatial segregation of signaling proteins within a raft facilitates efficient signaling along these pathways and pathogens themselves can subvert these carefully clustered signaling platforms for their own use [64,65]. For example, PAK1 (p21-activated kinase 1) and the GTPase Rac are clustered within lipid rafts in a manner that facilitates signaling processes involving these proteins [66]. Indeed, cholesterol-mediated disruption of lipid rafts at the plasma membrane has been suggested to interfere with both adenovirus and human immunodeficiency virus entry by disrupting the organization of relevant proteins and signaling molecules [63,67]. Similarly, defects in lipid raft trafficking have been shown to mislocalize the signaling GTPases (Rac1, Cdc42) necessary for proper formation of macropinosomes during *Salmonella* entry and to block the macropinocytic uptake of this pathogen [68].

The signaling pathways for macropinocytic pathogen uptake are thus another distinct example of a cellular signaling process that requires proper lipid raft regulation. This directly implies a role for myosin 1c in the regulation of these signaling pathways via its role in lipid raft recycling. Such a role for myosin 1c is supported by studies demonstrating that myosin 1c colocalizes with membrane ruffles at the lipid raft-rich entry sites of the bacterial pathogen *Salmonella enterica*, and that a knockdown of myosin 1c reduces the formation of macropinosomes and inhibits *Salmonella* invasion [35]. Myosin 1c is thus strongly implicated as a player in the signal transduction pathways governing pathogen entry by macropinocytosis (Fig. 1 (2)).

Thus, in summary, this section has discussed the suggestion that the newfound role for myosin 1c in lipid raft regulation makes this motor a general player in intracellular signaling pathways. Recent evidence specifically indicates lipid raft based functions for myosin 1c in the integrin-mediated pathways involved in cell spreading and migration, as well as in the signaling cascades involved in pathogen uptake by macropinocytosis. Given the widespread involvement of lipid rafts in cell signaling, these pathways are likely to be only a few of the cellular signaling pathways in which myosin 1c plays a lipid raft-based role.

## 4 Further roles for myosin 1c in cell signaling

In addition to the function of myosin 1c in specific signaling pathways linked to its role in lipid raft recycling, myosin 1c has also been suggested to play a more direct role in other intracellular signaling cascades by mediating the delivery of lipid raft associated signaling components or by acting as a direct player in signal transduction.

### 4.1 Myosin 1c in Neph1 signaling

The podocyte (visceral epithelial) cells of the kidneys characteristically extend foot-like, actin-based projections that wrap around the capillary network within the glomerulus. Blood passing through these capillaries is pressure filtered through the slits between these foot-like projections [69] (See Fig. 1 (3)). The formation of such actin-based foot projections at the podocyte membrane relies on a signaling transduction cascade involving the transmembrane proteins Neph1 and nephrin. In particular, activation of a nephrin–Neph1 complex by the Src family protein kinase Fyn results in recruitment of the adaptor proteins Nck (non-catalytic region of tyrosine kinase adaptor protein 1) and Grb2 (growth factor receptor-bound protein 2) that regulate actin polymerization via WASP proteins and the Arp2/3 complex [70].

Recently it has been shown that myosin 1c binds both Neph1 and nephrin and mediates their localization to outer membranes, and that myosin 1c depletion disrupts this localization and so the signaling pathways necessary to form the actin structures required for podocyte migration [28]. Arif et al. suggest that this involvement of myosin 1c in Neph1 signaling stems from the fact that myosin 1c recruits the Neph1–nephrin complex to lipid rafts for membrane delivery and signaling compartmentalization and may also be involved in the anchoring of the Neph1 complex at the plasma membrane.

### 4.2 Myosin 1c in tumor necrosis factor-alpha (TNF-alpha) induced insulin resistance

One of the leading risk factors for the development of type 2 diabetes is resistance to insulin-stimulated glucose uptake or ‘insulin resistance’ [71]. The development of insulin resistance in a given cell relies on a signaling pathway involving adipocytokine TNF-α activation of IκB kinase (IKK) and eventual phosphorylation of IRS-1 (insulin receptor substrate 1), which reduces the metabolic response to insulin [72,73] (See Fig. 1 (4)). The involvement of myosin 1c in this signaling pathway has been clearly demonstrated [74]. Myosin 1c binds to a subunit of IKK (nuclear factor κB essential modulator (NEMO)/IKK-γ) and is required for the translocation of this protein to its functional site at the plasma membrane. Overexpression of a dominant negative form of myosin 1c reduces the interaction between IKK and IRS-1 and reduces phosphorylation of IRS-1, suggesting clearly that the motor mediates TNF-α-induced down-regulation of IRS-1 and glucose uptake [74]. Myosin 1c may play a role in this signaling pathway by mediating lipid raft based transport of NEMO/IKK-γ or clustering of TNF-α pathway signaling molecules at the membrane. Alternatively, the myosin 1c-NEMO complex could play a direct functional role in the signaling cascade that mediates phosphorylation of IRS-1 [74].

### 4.3 Myosin 1c as a direct player in insulin-mediated signaling for glucose receptor transport

Glucose uptake into adipocyte and muscle cells involves insulin hormone-stimulated transfer of GLUT4 (glucose transporter 4) to the plasma membrane. GLUT4 is a glucose transporter that directly facilitates glucose uptake [75]. The transfer of GLUT4 occurs via a series of signaling cascades characteristically involving the serine/threonine kinase Akt [76]. The involvement of myosin 1c in the translocation of GLUT4 containing vesicles to the plasma membrane of both adipocytes and skeletal muscle cells is well established [24,77] (See Fig. 1 (5)). A recent study demonstrates that the role for myosin 1c in GLUT4 translocation is based on the direct involvement of this motor as a novel target in an insulin signaling pathway [78]. In particular, insulin stimulation activates the serine/threonine kinase CaMKII (Ca^2 +^/calmodulin-dependent protein kinase II) by increasing the calcium concentration beneath the plasma membrane. CaMKII then phosphorylates myosin 1c at Ser701, and myosin 1c binds a 14-3-3 regulatory protein. This results in an increase in the ATPase activity of myosin 1c, which may facilitate the docking or fusion of GLUT4 vesicles at the plasma membrane. This new myosin 1c-dependent signaling pathway is critical for proper GLUT4 transfer and the resulting uptake of glucose, as abolishment of this pathway using CaMKII inhibitors blocks insulin-stimulated glucose transport in adipocytes [78].

Myosin 1c is thus involved in cell signaling pathways not only via lipid raft regulation and protein anchoring/transfer, but also as a directly phosphorylated player in an insulin-mediated signaling cascade. In fact, the role of myosin 1c as an insulin-regulated 14-3-3 target in this pathway has led to the speculation that the motor may be phosphorylated by other Ca^2 +^/calmodulin-dependent protein kinases in other similar transduction pathways [79].

### 4.4 Myosin 1c in mechanical signal transduction in hair cells

Mechanical signal transduction or ‘mechanotransduction’ is a specialized type of cellular signaling event in which mechanical signals are converted into biochemical responses. In the hair cells of the inner ear, this signaling process permits the transfer of the mechanical vibrations passing through the ear canal into electrical stimuli that travel along neurons to signal the brain [80]. In particular, a mechanical sound stimulus causes the physical deflection of specialized microvilli termed stereocilia and so generates a change in tension that opens a series of tension-gated ion channels at the tips of the stereocilia [23]. The resulting influx of calcium ions through these ion channels induces a membrane depolarization that triggers a neurotransmitter release and so activates adjacent auditory nerve fibers [80] (See Fig. 1 (6)). This influx of calcium can occur on a short time scale (a few milliseconds or less before channel closure) or a longer time scale (more than 10 milliseconds before channel closure). These processes are termed fast and slow adaptation, respectively [81].

Myosin 1c has been regarded as a key player in mechanical signal transduction in hair cells for many years now. During slow adaptation, myosin 1c motors connected to the stereocilia membrane slide along actin filaments to reduce the tension on the gated ion channels caused by stereocilia displacement, thereby permitting channel closure and signal attenuation [23]. This tension-modulating role for myosin 1c in mechanical signal transduction is believed to be regulated by calcium, ATP, and the phosphatidylinositol 4,5-bisphosphate (PIP_2_) in the stereocilia membrane [82,83]. The finding that expression of mutant myosin 1c constructs in hair cells blocks not only slow adaptation but also fast adaptation suggests that the well-studied role for myosin 1c in slow adaptation may be complemented by a similar role in fast adaptation [84,85]. The overall importance of myosin 1c in this signaling process is confirmed by the observation that mutations in myosin 1c are linked to hearing loss in the human population [86].

This function for myosin 1c in mechanical signal transduction in the inner ear demonstrates that myosin 1c can play a role in cellular signaling directly based on its capabilities for tethering and force generation. Interestingly, preliminary evidence indicates that myosin 1c may also regulate signal transduction via ion channels in different body systems, as proper myosin 1c function is necessary for normal transport through sodium ion channels in collecting duct cells upon antidiuretic hormone (ADH) stimulation [29].

## 5 Myosin 1c in cell signaling and disease

Considerable evidence now indicates that defects in cellular signaling pathways are intimately linked to disease. For example, defects in the integrin signaling pathways mediating adhesion and migration have been implicated in rheumatoid arthritis, inflammatory bowel disease, and muscular dystrophy [87], while over expression of integrins is linked to tumor growth in many forms of cancer [88]. Similarly, defects in proper insulin signaling and the signaling pathways leading to insulin resistance are directly linked to the onset of type 2 diabetes, obesity, and Alzheimer's disease [89,90]. Furthermore, defects in Neph1 signaling pathways in podocytes have been linked to focal segmental glomerulosclerosis and membranous nephropathy [91,92], and defects in mechanical signal transduction in hair cells cause deafness [93].

The newly emerging importance of myosin 1c in each of these signaling pathways (and many more) thus implicates this motor as a target for drug-based treatment of many critical human diseases, from Alzheimer's disease to diabetes. For example, targeted reduction of functional myosin 1c levels could combat overexpression of insulin-mediated signaling pathways in tumor migration. In addition, therapeutic restoration of functional myosin 1c could restore proper mechanical signal transduction in deaf individuals. The amenability of myosin 1c to drug-based therapies is supported by a recent study showing that the motor activity and intracellular trafficking functions of myosin 1c can be inhibited by the small molecule pentachloropseudilin [94]. In short, the important role played by myosin 1c in cellular signaling makes this motor a valuable new drug target for the treatment of many diseases.

## 6 Conclusions

• The molecular motor myosin 1c is a prominent player in many signal transduction pathways throughout the cell, from the integrin-mediated pathways involved in cell migration to the Neph1 signaling pathways involved in actin organization in podocytes.
• The role played by myosin 1c in many of these pathways is based on the fact that this motor regulates the intracellular recycling of lipid rafts, which serve as important platforms for the organization and coordination of proper signaling throughout the cell.
• Myosin 1c also plays a more direct role in intracellular signaling, e.g. as a phosphorylated member of the signaling transduction pathways mediating GLUT4 trafficking and as a tension-based modulator of mechanical signal transduction in the inner ear.
• Defects in all of these signaling events cause distinct forms of human disease. As such, the role of myosin 1c as a new player in intracellular signaling makes this motor an important target for drug-based therapeutics.
